# Supplementary material for: Root cap cell corpse clearance limits microbial colonization in Arabidopsis thaliana
Source: eLife. 2024 Nov 12;13:RP96266. doi: 10.7554/eLife.96266 (PMC11556792; doi:10.7554/eLife.96266)
Supplement: Figure 1—source data 1. [file elife-96266-fig1-data1.zip › Figure 1-Source Data 1.pdf]

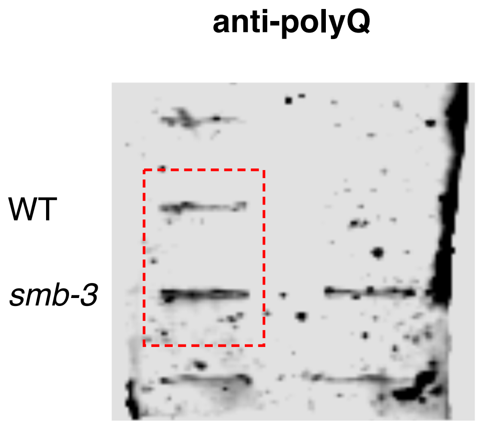

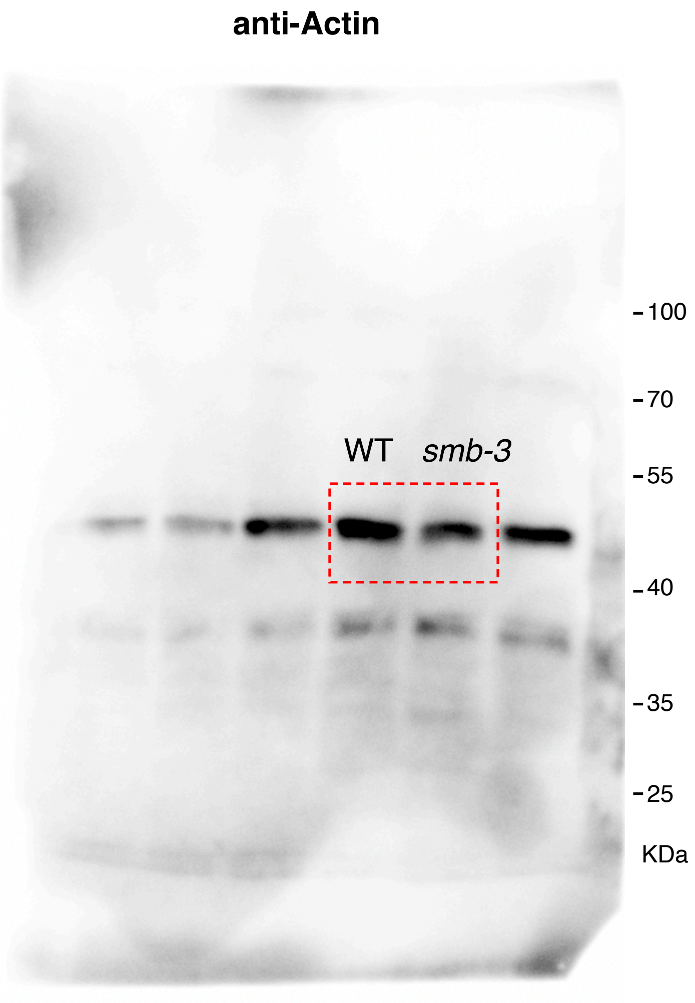

Figure 1 - Source Data 1. Original blots corresponding to Figure 1, panel G. This includes both the filter trap and Western blot. Cropped sections used in the main Figure 1 are highlighted with a dotted red rectangle. Relevant bands are labeled for clarity.
