# Supplementary figures and images for: Root cap cell corpse clearance limits microbial colonization in Arabidopsis thaliana
Source: eLife. 2024 Nov 12;13:RP96266. doi: 10.7554/eLife.96266 (PMC11556792; doi:10.7554/eLife.96266)

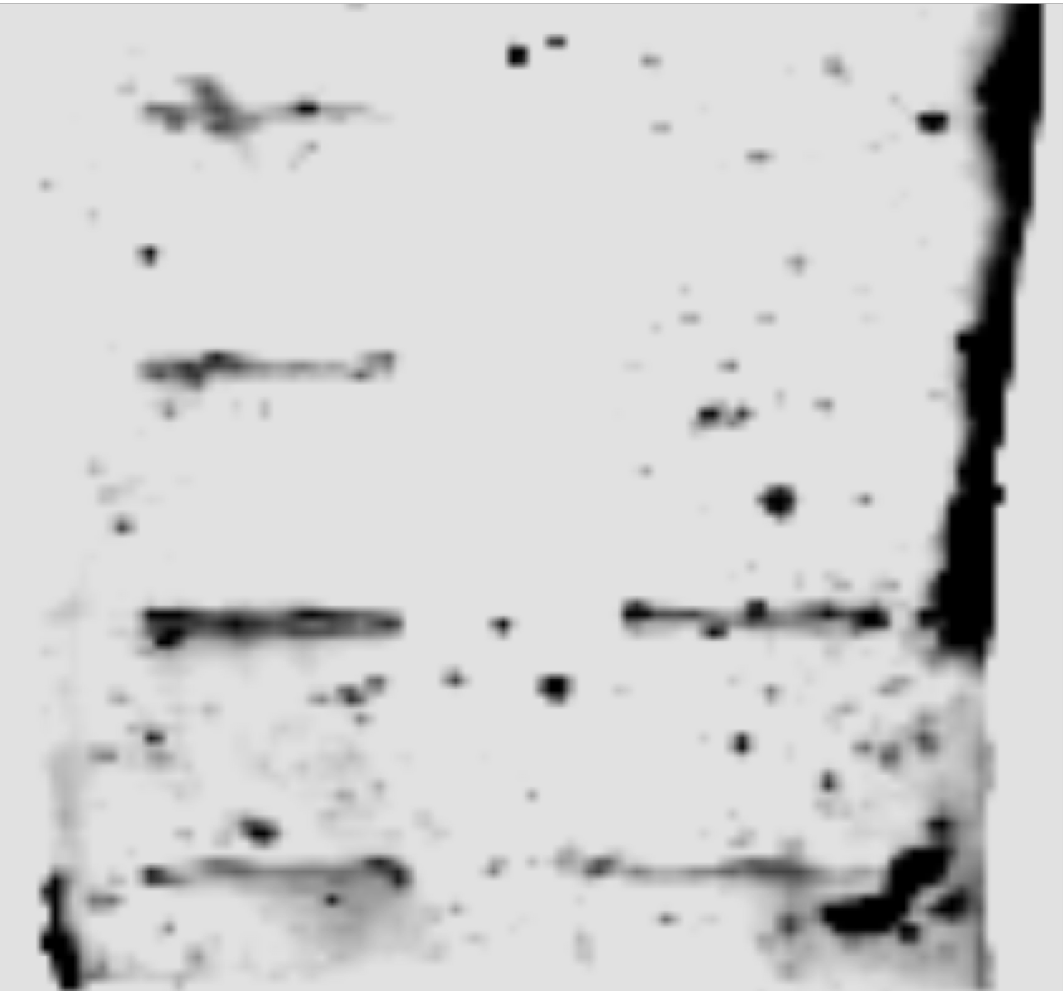

Supplement: Figure 1—source data 2. [file elife-96266-fig1-data2.zip › Figure 1-Source Data 2/Figure 1 -Source Data 2_FiltertrapRaw.tiff]

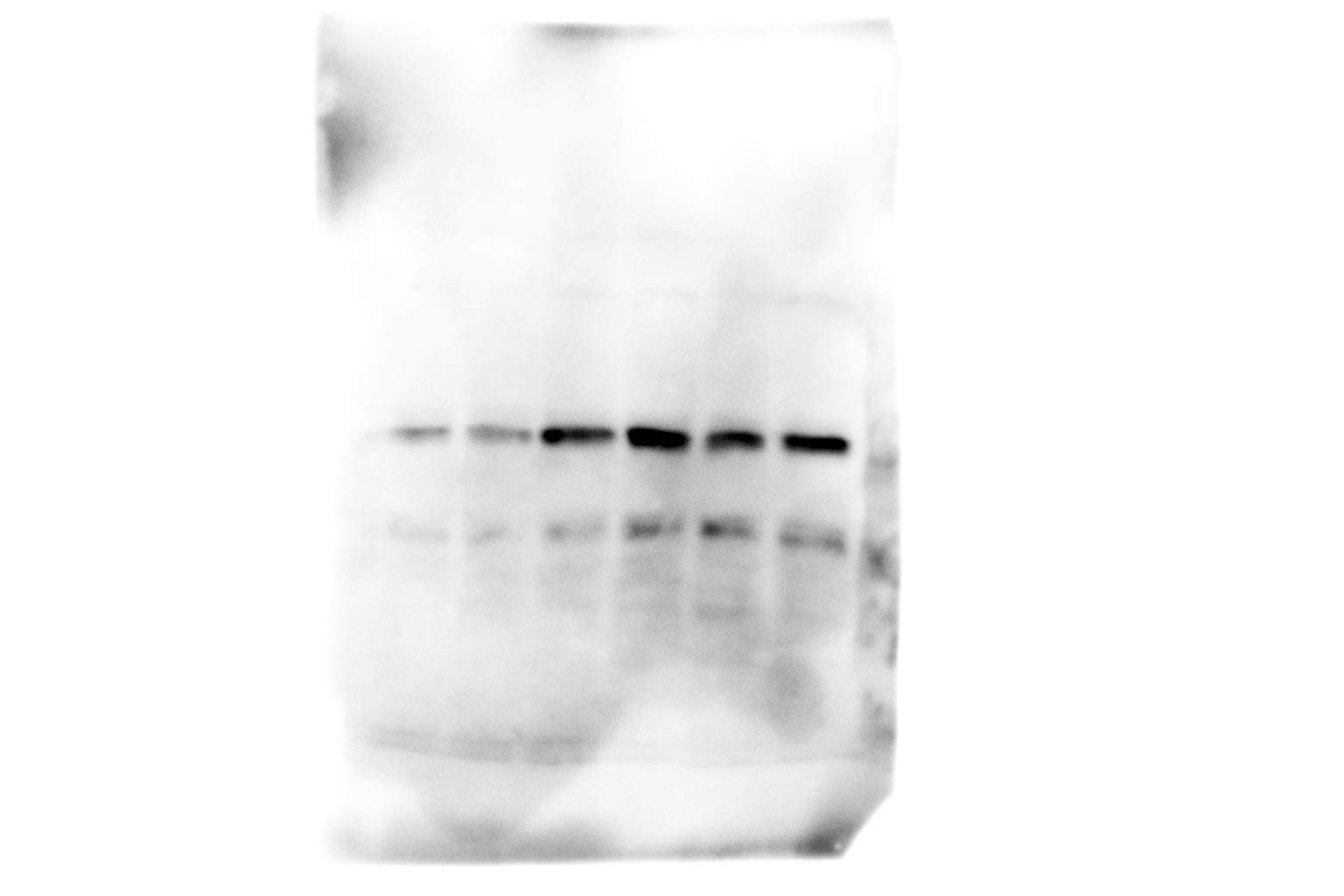

Supplement: Figure 1—source data 2. [file elife-96266-fig1-data2.zip › Figure 1-Source Data 2/Figure 1-Source Data_2_SDSPageRaw.tif]
